# Potential utility of eGFP-expressing NOG mice (NOG-EGFP) as a high purity cancer sampling system

**DOI:** 10.1186/1756-9966-31-55

**Published:** 2012-06-06

**Authors:** Kentaro Shima, Masamichi Mizuma, Hiroki Hayashi, Kei Nakagawa, Takaho Okada, Naoaki Sakata, Noriyuki Omura, Yo Kitamura, Fuyuhiko Motoi, Toshiki Rikiyama, Yu Katayose, Shinichi Egawa, Naoto Ishii, Akira Horii, Michiaki Unno

**Affiliations:** 1 Division of Hepato-Biliary-Pancreatic Surgery, Tohoku University Graduate School of Medicine, 1–1 Seiryo-machi Aoba-ku, Sendai, 980-8574, Japan; 2 Division of Integrated Surgery and Oncology, Department of Surgery, Tohoku University Graduate School of Medicine, Sendai, Japan; 3 Department of Microbiology and Immunology, Tohoku University Graduate School of Medicine, Sendai, Japan; 4 Department of Molecular Pathology, Tohoku University Graduate School of Medicine, Sendai, Japan

**Keywords:** NOG-EGFP mouse, Xenograft, Cancer, Stromal cell, Separation

## Abstract

**Purpose:**

It is still technically difficult to collect high purity cancer cells from tumor tissues, which contain noncancerous cells. We hypothesized that xenograft models of NOG mice expressing enhanced green fluorescent protein (eGFP), referred to as NOG-EGFP mice, may be useful for obtaining such high purity cancer cells for detailed molecular and cellular analyses.

**Methods:**

Pancreato-biliary cancer cell lines were implanted subcutaneously to compare the tumorigenicity between NOG-EGFP mice and nonobese diabetic/severe combined immunodeficiency (NOD/SCID) mice. To obtain high purity cancer cells, the subcutaneous tumors were harvested from the mice and enzymatically dissociated into single-cell suspensions. Then, the cells were sorted by fluorescence-activated cell sorting (FACS) for separation of the host cells and the cancer cells. Thereafter, the contamination rate of host cells in collected cancer cells was quantified by using FACS analysis. The viability of cancer cells after FACS sorting was evaluated by cell culture and subsequent subcutaneous reimplantation in NOG-EGFP mice.

**Results:**

The tumorigenicity of NOG-EGFP mice was significantly better than that of NOD/SCID mice in all of the analyzed cell lines (*p* < 0.01). Sorting procedures enabled an almost pure collection of cancer cells with only slight contamination by host cells. Reimplantation of the sorted cancer cells formed tumors again, which demonstrated that cell viability after sorting was well maintained.

**Conclusions:**

This method provides a novel cancer sampling system for molecular and cellular analysis with high accuracy and should contribute to the development of personalized medicine.

## Introduction

Cancer xenograft models of immunodeficient mice are widely applied in various cancer research areas. Recently, xenografted human tumors are commonly used for preclinical drug testing, including biomarker discovery. [1,2] It has been reported that there is a close correlation between the effects in xenografts and clinical outcomes, in terms of both drug resistance and sensitivity. [3] An eventual goal of such preclinical studies using mouse xenograft models is the realization of personalized medicine. Molecular analyses using clinical specimens or xenografted tumors are essential in research for personalized medicine, and high purity samples of sufficient volume are necessary for precise analyses. In general, mouse xenografts are superior to clinical specimens because of the abundance and renewability of the tumor samples.

Tumors consist of two components, i.e. cancer cells and stroma. Stromal cells derived from murine cells within the xenografted tumors. Even though tumor tissue acquired from patients is transplanted, human stromal cells are ultimately replaced by murine stromal cells [4]. Accordingly, contamination by stromal cells hinders precise analyses of cancer cells using tumor tissue. Although stromal cells need to be removed from tumor tissue as much as possible to obtain accurate results, it is still technically difficult to collect high purity cancer cells without contamination by stromal cells. As technologies of comprehensive analyses (e.g., high-resolution microarray, next-generation sequencing and proteomics) are progressing rapidly, high purity samples uncontaminated by stromal cells are necessary for such advanced technology. Therefore, it is very important to establish a method of separating cancer cells and stromal cells clearly and collecting cancer cells uncontaminated by stromal cells.

On the other hand, athymic nude mice, nonobese diabetic/severe combined immunodeficiency (NOD/SCID) mice or NOD.Cg-*Prkdc*^*scid*^*Il2rg*^*tm1Sug*^*/ShiJic* (NOG) mice are routinely used for mouse xenograft models of cancer. Among these types of mice, NOG mice show the most severe immunodeficient state. Machida and colleagues have reported that NOG mice have higher susceptibility to xenografted tumors than other immunodeficient mice [5]. Thus, NOG mice are very useful for the transplantation of tumor tissue.

In 2008, Niclou and colleagues reported that NOD/SCID mice with ubiquitous expression of enhanced green fluorescent protein (eGFP) were useful for the clear separation of tumor cells and mouse stromal cells in subcutaneous xenografted tumors by fluorescence activated cell sorting (FACS), and demonstrated that the contamination by stromal cells after the removal of eGFP-expressing cells was slight. [6] Meanwhile, Suemizu et al. generated NOG mice expressing eGFP ubiquitously (NOG-EGFP) and clarified that NOG and NOG-EGFP mice have equivalent immunodeficient states. [7] However, there are no reports to study cancer xenograft of NOG-EGFP mice.

In this study, we hypothesized that NOG-EGFP mice are potentially useful for the collection of cancer cells without contamination by stromal cells and would also have the advantage of easy engraftment. Here we compare the tumorigenicity between NOG-EGFP and NOD/SCID mice and show the degree of contamination by stromal cells after removal of eGFP-expressing cells in the xenografted tumors of NOG-EGFP mice by FACS. Furthermore, we demonstrate the viability of the collected cancer cells by cell culture and subsequent inoculation.

## Materials & methods

### Ethics

All animal experiments conformed to the guidelines of the Institutional Animal Care and Use Committee of Tohoku University and were performed in accordance with the Guide for the Care and Use of Laboratory Animals of Tohoku University. The protocol was approved by the Ethics Review Committee of Tohoku University.

### Animals

6 week-old female NOG-EGFP (formally, NOD.Cg-Prkdc^scid^Il2rg^tm1Sug^Tg (Act-eGFP) C14-Y01-FM1310sb/ShiJic) mice and NOG mice were kindly provided by Central Institute for Experimental Animals (Kawasaki, Japan). NOD/SCID mice were purchased from CLEA Japan, Inc. (Tokyo, Japan). Female heterozygous NOG-EGFP mice were mated with male NOG mice in order to breed the NOG-EGFP mice under the permission of Central Institute for Experimental Animals. Since their offspring were NOG mice or NOG-EGFP mice, the fluorescence of NOG-EGFP mice was confirmed by a hand-held UV lamp (COSMO BIO, Tokyo, Japan). Thereafter, NOG-EGFP mice were used in the experiments. The animals were housed under pathogen-free conditions on a 12-hour light cycle and with free access to food and water.

### Cell culture

Human pancreatic cancer cell lines (MIA Paca2 and AsPC-1) and human cholangiocarcinoma cell lines (HuCCT1 and TFK-1) were obtained from the Cell Resource Center for Biomedical Research of Tohoku University. HuCCT1, TFK-1 and AsPC-1 were cultured in RPMI-1640 media (Sigma-Aldrich, MO, USA) with 10% heat-inactivated fetal bovine serum (FBS) (SAFC Biosciences, MO, USA) and 1% penicillin/streptomycin (P/S) (Gibco/Life Technologies, CA, USA) at 37°C in an atmosphere of 5% CO_2_ and 95% air. Dulbecco modified Eagle medium (DMEM) (Gibco/Life Technologies) was used for culture of MIA PaCa2 cells.

### Image acquisition

We confirmed that organs and cells obtained from NOG-EGFP mice could be fluorescently visualized. In detail, after euthanizing NOG-EGFP mice, internal organs were placed on a tray and imaged using an IVIS® Spectrum system (Caliper Life Sciences, MA, USA). Skin fibroblasts of NOG-eGFP mice were cultured in RPMI-1640 media with 10% FBS and 1% P/S. Subsequently, cultured fibroblasts on dishes were visualized using a Keyence BZ-9000 fluorescence microscope (Keyence Corporation, Osaka, Japan).

### Cell transplantation in NOG-EGFP and NOD/SCID mice

5 × 10^5^ cells in a total volume of 100 μl media were injected subcutaneously into each side of the lower back of 6-8-week-old NOG-EGFP mice and NOD/SCID mice. Tumor size was measured with digital calipers (A&D, Tokyo, Japan) twice a week. Tumor volume was determined using the following formula [8]:

(1) $$\text{tumor volume=}\left[length\times width^{2}\right]/2$$

### Patient-derived cancer xenografts

Resected specimens of pancreatic cancer tissue were cut into 2–3mm^3^ pieces in antibiotic-containing RPMI-1640 media. Under anesthesia with pentobarbital (Abbott Laboratories, IL, USA), and sevoflurane (Maruishi Pharmaceutical, Osaka, Japan), the pieces of the tumors were implanted subcutaneously into each side of the lower back in 6–8–week-old female NOG-EGFP mice. Tumors were harvested upon reaching a volume of 1,500 mm^3^ and provided for immunohistochemistry.

### Immunohistochemistry

Subcutaneous tumors of NOG-EGFP xenografts were fixed in 10% formalin before embedded in paraffin. After blocking, immunohistochemistry for eGFP was performed using a rabbit anti-GFP (ab290, Abcam, MA USA) at a dilution of 1:1000 incubated for 1hour at 25°C. A horseradish peroxidase (HRP)-conjugated goat anti-rabbit IgG (Nichirei Biosciences, Tokyo, Japan) was used as the secondary antibody. Peroxidase visualization was done using 3,3'-Diaminobenzidine (DAB). All techniques including H&E staining were performed by Animal Pathology Platform, Biomedical Research Core of Tohoku University Graduate School of Medicine.

### Cell sorting and phenotyping of murine stromal cells

TFK-1 xenografts were used in this experiment. Freshly isolated subcutaneous tumors of NOG-EGFP mice were dissociated by mincing the tissue with scalpels, followed by incubation in RPMI-1640 media containing collagenase (Worthington Biochemical, NJ, USA) for 30 min at 37°C. After incubation, the cell suspension was filtered through a 100-μm cell strainer. The cells were resuspended in phosphate buffered saline (PBS) and sorted on a fluorescence-activated cell sorter (FACS Aria ^TM^ II Cell Sorter, BD Biosciences, Erembodegem, Belgium) on the basis of single-cell viability and the presence of GFP. For immunophenotyping, cells were incubated for 30 min at room temperature with conjugated antibodies against mouse CD31, CD90, CD49b, CD14, CD11c (CD31: 561410, CD90: 553007, CD49b: 553858, CD14: 560636 and CD11c: 560583, BD Biosciences) or conjugated isotype controls (APC-Cy^TM^7 (Rat IgG1, κ)-560534, Alexa-Flour700 (Hamster IgG, λ1): 560555, APC (Rat IgG2a, κ): 53932, PE (Rat IgM, κ): 553943, PE-Cy^TM^7 (Rat IgG2a, κ): 552867, BD Biosciences), as previously reported [6] . Analyses were performed on a FACS Aria ^TM^ II Cell Sorter (BD Biosciences).

### Viability of sorted cancer cells

Xenografted tumors of TFK-1 cells in NOG-EGFP mice were harvested and separated into cancer cells and stromal cells by FACS as described above. Collected TFK-1 cells were cultured on dishes and subsequently reimplanted in NOG-EGFP mice. In order to confirm the effect of removal of eGFP-expressing cells, the subcutaneous tumors of TFK-1 cells were provided for primary cell culture without FACS sorting as a control.

### Statistical analysis

Data were presented as the mean ± S.E. Statistical significance was determined by Mann–Whitney *U* test performing using GraphPad Prism for Windows version 5.02. Differences between experimental groups were considered significant when the *p*-value was <0.05.

## Results

### Confirmation of eGFP expression in NOG-EGFP mice

**Figure 1:**
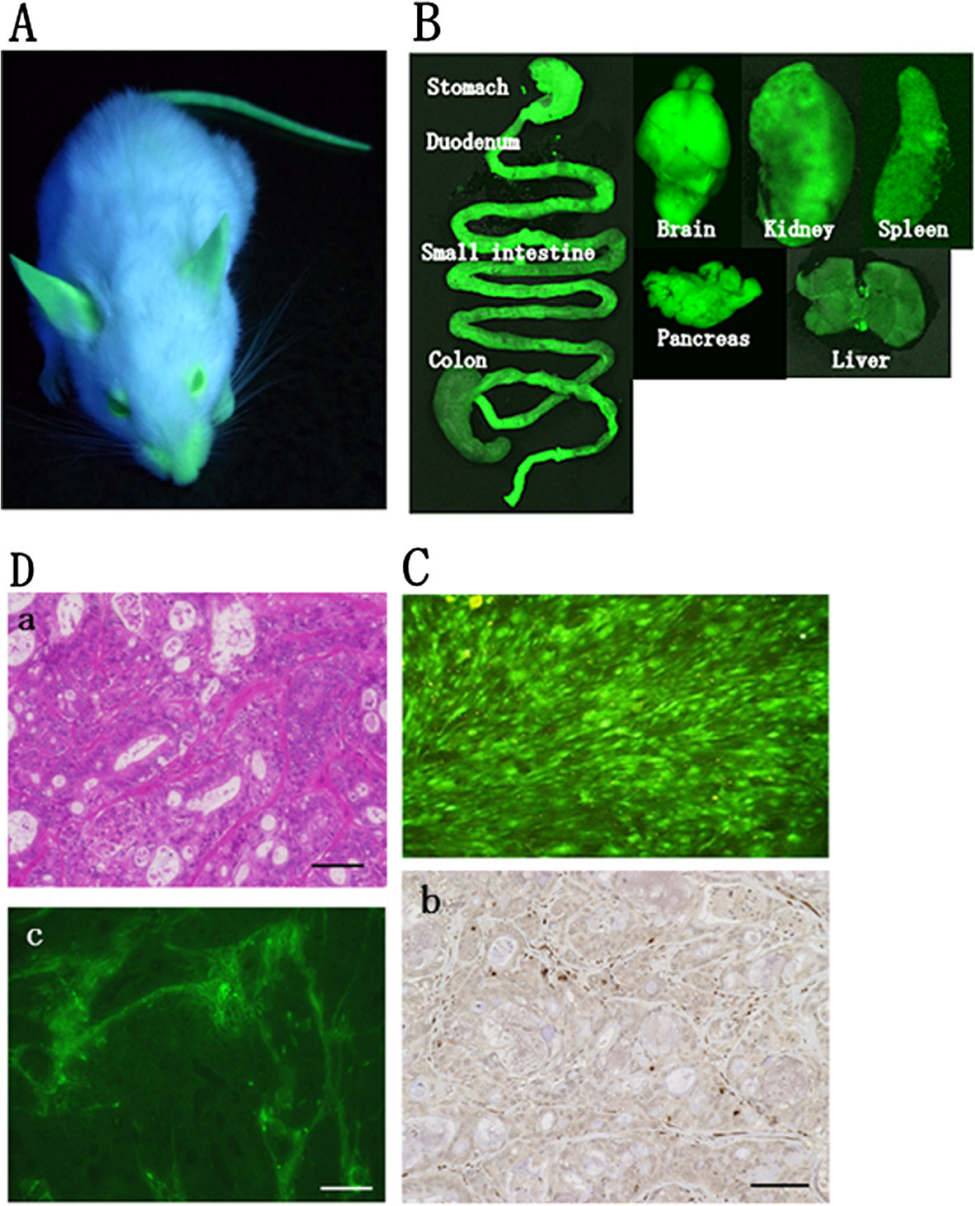
**Confirmation of eGFP expression in NOG-EGFP mice.A**) NOG-EGFP mice were fluorescently visualized under a hand-held UV lamp. **B**) Representative photos of internal organs of NOG-EGFP mice. The fluorescence was detected in all internal organs with IVIS® spectrum system. **C**) Skin fibroblasts of NOG-EGFP mice cultured on the dishes were fluorescent under the fluorescence microscope. **D**) Histology of patients-derived pancreatic cancer xenografts in NOG-EGFP mice. **D-a**) H&E staining. **D-b**) immunohistochemistry of the anti-eGFP antibody. eGFP-expressing cells are seen in the stroma. **D-c**) eGFP positive cells visualized under the fluorescence microscope are seen in the stroma, concordant with of Figure 1Db.

Green fluorescence was detected in the NOG-EGFP mice by a hand-held UV lamp (Figure 1A). Almost all internal organs showed green fluorescence in the imaging instrument (Figure 1B). The fluorescence of skin fibroblasts was visible using a fluorescence microscope (Figure 1C). Histological findings revealed eGFP-expressing cells (shown as DAB-positive cells in Figure 1Db and fluorescent cells in Figure 1Dc) in the stroma of the xenografted tumors, whereas cancer cells did not show eGFP expression (Figure 1Db-c). Based on the findings mentioned above, expression of eGFP on NOG-EGFP mice was confirmed.

### Comparison of tumorigenic potential between NOG-EGFP and NOD/SCID mice

**Figure 2:**
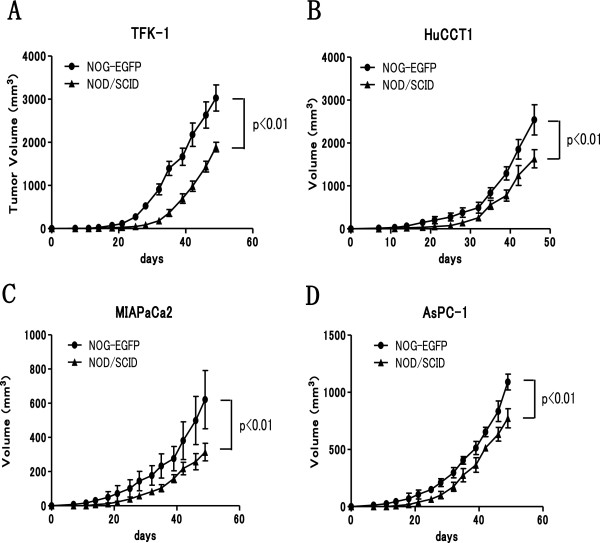
**Tumorigenicity was compared between NOG-EGFP mice and NOD/SCID mice using the pancreato-biliary cancer cell lines.A**) TFK-1, **B**) HuCCT1, **C**) MIAPaCa2 and **D**) AsPC-1. A total of 5.0 × 10^5^ cells was injected into each mouse (n = 6). ** denotes *P* < 0.01. NOG-EGFP mice showed a significantly higher tumorigenic potential than that of NOD/SCID mice in all cell lines ( *p* < 0.01).

Human pancreatic cancer cell lines (MIA PaCa2 and AsPC-1) and human cholangiocarcinoma cell lines (TFK-1 and HuCCT1) were inoculated into NOG-EGFP mice and NOD/SCID mice for comparison of the tumorigenic potential. The tumorigenic potential of the NOG-EGFP mice was significantly superior (p < 0.01) to that of the NOD/SCID mice in all cell lines (Figure 2A-D).

### Separation of cancer cells and stromal cells

**Figure 3:**
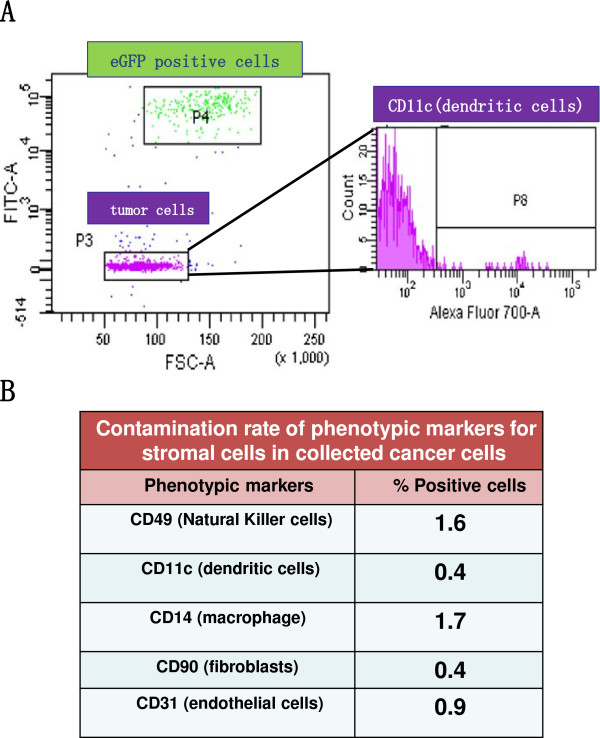
**The FACS analysis was performed after single-cell suspension obtained by enzymatic dissociation from xenografted tumors of NOG-EGFP mice.A**) Two subpopulations indicating the cancer cells and eGFP-expressing cells were clearly distinguished. The collected cancer cells were dyed with phenotypic markers to evaluate the contamination rate of host cells in the collected cancer cells. Results of CD11c are shown as representative data of the phenotypic markers. **B**) The contamination rates of the phenotypic markers for murine stromal cells among the collected cancer cells are summarized as a table; note that only a few host cells are contaminated.

A single-cell suspension was obtained by enzymatic dissociation from the xenografted tumors of TFK-1 cells. The cancer cells and the GFP-expressing cells were sorted using FACS. FACS analysis showed two subpopulations clearly enabling us to separate the cancer cells and the GFP-expressing cells (Figure 3A). Then, the subpopulation of cancer cells was collected for phenotyping of murine stromal cells. CD31, CD90, CD49b, CD14 and CD11c are specific markers suggesting the existence of endothelial cells, fibroblasts, natural killer cells, macrophage and dendritic cells, respectively. The percentages of mouse CD31, CD90, CD49b, CD14 and CD11c positive cells in the subpopulation of the cancer cells were almost below the detection level (0.9%: CD31; 0.4%: CD90; 1.6%: CD49b; 1.7%: CD14 and 0.4%: CD11c (Figure 3B). These results demonstrated that the accuracy of the separation of the cancer cells and the host cells in this study was the same as in the previous report [6].

### Cell viability after FACS sorting

**Figure 4:**
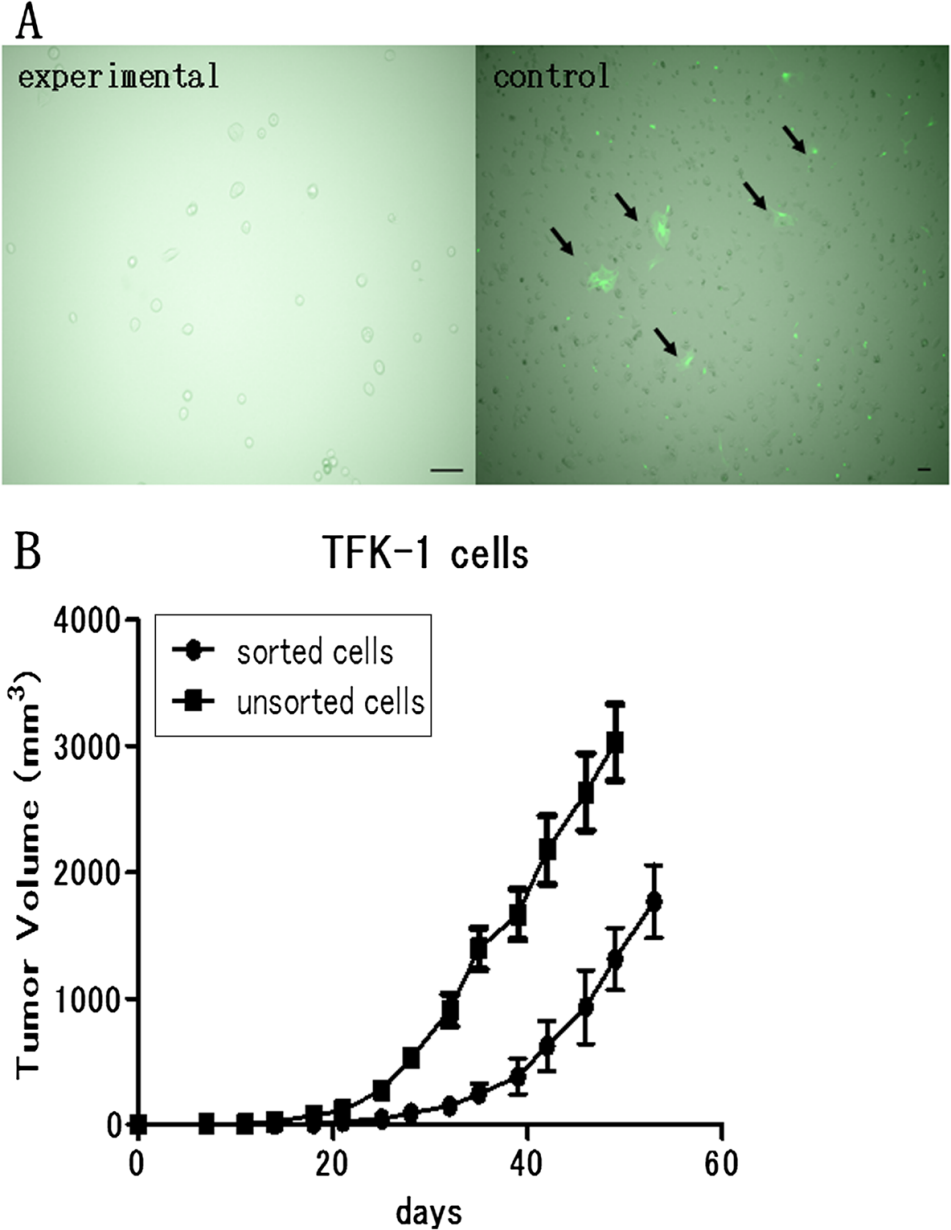
**In order to determine the cell viability, the cancer cells were cultured on dishes after FACS sorting and subsequently reimplanted into NOG-EGFP mice.A**) Left panel (experimental): The fluorescent cells were invisible among the collected cancer cells cultured on the dishes under the fluorescent microscope. Right panel (control): Directly cultured cells from the xenografted TFK-1 tumors. Fluorescent cells were detectable in some areas under the fluorescent microscope. Black arrows indicate eGFP-expressing cells. **B**) TFK-1 cells cultured after FACS sorting were able to grow in the NOG-EGFP mice. Tumorigenicity of the sorted TFK-1 cells was directly compared with that of the unsorted TFK-1 cells shown in Figure 2A. A total amount of 5.0 × 10^5^ cells was injected into each mouse (n = 6).

Cancer cells collected from TFK-1 xenografts of NOG-EGFP mice by FACS were able to grow on the dishes (Figure 4A). Few fluorescent cells were detectable among the collected cancer cells (experimental) on the dishes, whereas the unsorted cancer cells (control) showed a mixture of fluorescent and non-fluorescent cells (Figure 4A). These results demonstrated that FACS sorting could completely separate cancer cells and stromal cells. Subsequent reimplantation after cell culture showed that the sorted cancer cells had tumorigenic ability (Figure 4B). Since the period from inoculation to beginning of growth was longer in the sorted TFK-1cells than in the unsorted TFK-1 cells (Figure 4B), the viability of the sorted cells might have been lower than that of the unsorted cells.

## Discussion

The aim of the present study was to develop methods for separating mice-xenografted human cancer cells from host cells by FACS with minimal amount of contamination and also to maintain the cell viability for subsequent analyses. For this purpose, we have developed techniques that employ NOG-EGFP mice.

To date, fluorescent immunodeficient mice, i.e. GFP nude mice [9], NOD/SCID EGFP mice [6] and NOG-EGFP mice [7], have been established. The previous reports showed that fluorescent mice were very useful to study the details of tumor-stroma interaction [10-12]. Recently, Niclou and colleagues reported the almost complete separation of cancer cells and host cells using xenografted tumors of a glioma cell line in NOD/SCID EGFP mice. Based on this report, we evaluated the contamination rate of murine stromal cells among each cell type collected cancer cells. Our results showed similar contamination rates to those of the previous report and suggest that fluorescent mice would be very useful for the separation of cancer cells from host cells. However, the purity of the separation might be different in tumor type and implantation site since content rate of stromal cells varies in them. Further studies including orthotopic models of several organs and use of other tumor types are needed to evaluate the purity of separation. We also demonstrated that that sorted cancer cells were able to grow *in vitro* and *in vivo*. One of the advantages is that the tumor cells start to grow significantly earlier in NOG-EGFP mice than in NOD/SCID mice. Our present results provide a novel way of employing of collected cancer cells for to various subsequent analyses. In the report of the NOD/SCID EGFP xenografts, cancer cells labeled with another type of fluorescence were used for the separation study [6]. The present study suggests that fluorescent labeling of cancer cells is not necessary for the separation of cancer cells and host cells.

On the other hand, this method is applicable for the collection of not only cancer cells but also stromal cells. The methodology using fluorescent mouse xenografts might usefully contribute to studies of cancer stromal cells.

In conclusion, NOG-EGFP has high potential utility for complete separation of cancer cells and stromal cells with minimal contamination, if any, from xenografted tumors. Further studies are needed to establish a solid methodology for the separation and collection of stromal/cancer cells, and the use of NOG-EGFP mice for this is very promising.

## Competing of interests

All of the authors declare no potential conflicts of interest.

## Authors’ contributions

KS, MM and NO designed research. KS performed the research. YK technically supported the experiments of the flow cytometry. NI contributed to the animal experiments. KS, MM, HH, KN, TO and NS analyzed data. KS and MM wrote the paper. MM and NS edited the manuscript. FM, TR, YK, SE, NI AH and MU reviewed the manuscript. MU integrated the entire study. All authors read and approved the final manuscript.

## Grant Support

This work was supported by Japan Society for the Promotion of Science Grant-in-Aids for Young Scientists (B: 23791512) (HH), (B: 23791515) (TO), (B: 23791514) (MM).

## References

[B1] Garber K From human to mouse and back: ‘tumorgraft’ models surge in popularity J Natl Cancer Inst 2009 101 6 8 19116380 10.1093/jnci/djn481

[B2] Walter K Eshleman J Goggins M Xenografting and harvesting human ductal pancreatic adenocarcinomas for DNA analysis Methods Mol Med 2005 103 103 111 15542900 10.1385/1-59259-780-7:103

[B3] Hidalgo M Bruckheimer E Rajeshkumar NV A pilot clinical study of treatment guided by personalized tumorgrafts in patients with advanced cancer Mol Cancer Ther 2011 10 1311 1316 10.1158/1535-7163.MCT-11-0233 21673092 PMC4629061

[B4] Hahn SA Seymour AB Hoque AT Allelotype of pancreatic adenocarcinoma using xenograft enrichment Cancer Res 1995 55 4670 4675 7553647

[B5] Machida K Suemizu H Kawai K Higher susceptibility of NOG mice to xenotransplanted tumors J Toxicol Sci 2009 34 123 127 10.2131/jts.34.123 19182442

[B6] Niclou SP Danzeisen C Eikesdal HP A novel eGFP-expressing immunodeficient mouse model to study tumor-host interactions FASEB J 2008 22 3120 3128 10.1096/fj.08-109611 18495755 PMC2518261

[B7] Suemizu H Yagihashi C Mizushima T Establishing EGFP congenic mice in a NOD/Shi-scid IL2Rg(null) (NOG) genetic background using a marker-assisted selection protocol (MASP) Exp Anim 2008 57 471 477 10.1538/expanim.57.471 18946184

[B8] Euhus DM Hudd C LaRegina MC Johnson FE Tumor measurement in the nude mouse J Surg Oncol 1986 31 229 234 10.1002/jso.2930310402 3724177

[B9] Yang M Reynoso J Jiang P Li L Moossa AR Hoffman RM Transgenic nude mouse with ubiquitous green fluorescent protein expression as a host for human tumors Cancer Res 2004 64 8651 8656 10.1158/0008-5472.CAN-04-3118 15574773

[B10] Fukumura D Xavier R Sugiura T Tumor induction of VEGF promoter activity in stromal cells Cell 1998 94 715 725 10.1016/S0092-8674(00)81731-6 9753319

[B11] Duda DG Fukumura D Munn LL Differential transplantability of tumor-associated stromal cells Cancer Res 2004 64 5920 5924 10.1158/0008-5472.CAN-04-1268 15342367

[B12] Yang M Li L Jiang P Moossa AR Penman S Hoffman RM Dual-color fluorescence imaging distinguishes tumor cells from induced host angiogenic vessels and stromal cells Proc Natl Acad Sci U S A 2003 100 14259 14262 10.1073/pnas.2436101100 14614130 PMC283579

